# Vaccine Confidential A Conversation with Donald G. McNeil Jr.

**DOI:** 10.4269/ajtmh.19-0427

**Published:** 2019-07-08

**Authors:** Claire Panosian Dunavan

**Affiliations:** University of California; Los Angeles, California; E-mail: cpanosian@mednet.ucla.edu

In 2006, a series about diseases on the brink of eradication ran in The New York Times, winning several awards. That November, one of the authors, Donald G. McNeil Jr., attended his first ASTMH conference. “I keep coming back,” McNeil said. “It covers many diseases, so I learn a lot. There’s always something based on recent news. And trop med people are just fun: where else do you hear doctors describe crossing the Zambezi up to their necks in crocodiles?”

McNeil began his career at The Times as a copy boy straight out of college, but was not assigned to cover global health until 2002. Nonetheless, he cut his teeth on HIV/AIDS while working in South Africa in the 1990s. After a stint in Paris, he returned to New York and was offered a job in science news. “You’ve already got two MDs covering health,” he argued to then-editor Cornelia Dean. “Why don’t I cover diseases that poor people die of?” She agreed to the experiment.

McNeil has now reported on everything from anthrax to Zika, but vaccine-preventable diseases are a recurring focus. In May 2019, he agreed to an email interview with AJTMH about covering vaccine controversies.

**Over the last 20 years, how many articles about vaccines have you reported and written? Please highlight some of the stand-outs and what they taught you.**

[I’ve written] hundreds, if you include everything I cover from measles and flu shots to the hunts for elusive HIV and malaria vaccines.

For me, the standouts were some I wrote upon joining the science staff in 2002. I was asked to cover vaccine refusals.

I went to Vashon Island in Washington to interview parents about why they refused. I heard their concerns — but also met parents whose kids got tetanus or who infected babies with pertussis. That made it clear how dangerous refusal can be.

For $75, I joined a New Jersey church created by chiropractors to grant religious exemptions, and learned that the anti-vax movement is centuries old, rooted in repugnance for variolation, cowpox vaccine, and germ theory itself.

I also covered the trail of polio cases from northern Nigeria to Mecca, and revealed that pilgrims were spreading it. That infuriated the Saudis — the government paper accused me of “blackening the hajj.” But after polio spread from Mecca to Indonesia and Yemen, I think my coverage was part of the reason the Saudis started vaccinating all pilgrims.

I also covered the Bush administration’s failed effort to vaccinate millions of Americans against smallpox in the lead-up to the Iraq war. That taught me that a few vaccines actually are lethal to some recipients and how carefully risks must be weighed. (I wish I’d had more clout as a medical writer then. Maybe The New York Times would have shouldered less blame for helping start that war. Given what I know now, it was obvious that Saddam had no weaponized smallpox. He would have had to immunize his entire army, the scars would have been obvious, and I could have asked someone — Kurdish journalists, for example — if his army was newly vaccinated.)

**When did you become aware that the anti-vax movement was more than a fringe-y backlash? Do you connect dots between religious extremism and anti-vax philosophies?**

I still think it’s fringe. More than 90 percent of Americans vaccinate. So do the vast majority of Orthodox Jews, Amish, and so on — but there are pockets of refusers.

I don’t think of it as truly based on religion at all. The Taliban in Afghanistan are officially pro-vaccine, just like the Vatican, the Rabbinical Court in Jerusalem, and top Islamic scholars. “Religious” objections to things like porcine gelatin or cell lines from aborted fetuses are shams ginned up by the anti-vaxxers to scare devout parents.

I see the root problem as the “I won’t do it, and you can’t make me” attitude. There are always some people who don’t like being ordered: to pay taxes, to wear seat belts, to serve in the army, whatever. In this case, they refuse even if it protects their children from gruesome deaths.

I think we need to keep this measles outbreak — and vaccine resistance in general — in perspective. As I write this (in late May), we’re having the worst measles year since 1994, but it’s still less than 1,000 cases and zero deaths so far. We have 37,000 car crash deaths a year, 42,000 opioid overdose deaths, 40,000 gun deaths, and so on. Avoiding measles is easy, so what will make vaccination rates rise again — in my cold-blooded opinion — will be the very public death of one cute child. No one cared about hundreds of Syrian refugees drowning until that boy in a red shirt, Alan Kurdi, was photographed face down on a Turkish beach.

A less cynical way to change it is via legislation, as was done in California.^1^ No matter how much some people kick and scream, government actually can make you do some things for the good of all, like pay taxes.

I don’t worry that we will go back to the old days. Women once routinely had five children, assuming one or two would die. Now most have two. They can’t afford to be blithely fatalistic.

**Please discuss the curious political coalition now seen in the U.S.—i.e., right and left-leaning parents aligned against vaccines.**

The Jenny McCarthy crowd^2^ is now aligned with the Tea Party “Texans for Vaccine Choice” crowd. That doesn’t surprise me. I went from a Jesuit high school to UC Santa Cruz; I’ve always seen the far right and left as doppelgangers. Very few people actually reject all science, but they pick the science that matches their politics. For example, I think most liberal anti-vaxxers accept global warming and the religious right accepts gravity and thermodynamics while rejecting evolution. At one time, if you were a good Catholic, you had to believe that people rose from the dead and the sun revolved around the earth; now you only have to believe the former.

**Are you concerned that a backlash against current, proven vaccines could extend to new vaccines or other public health interventions that may be needed in a crisis?**

Not necessarily. Some vaccine skepticism is based on actual malfeasance or lying by health officials and industry executives who should know better. The Cutter polio vaccine incident^3^ was a disaster, as was Dengvaxia’s rollout in the Philippines.^4^ The whole-cell pertussis vaccine really did have scary side effects. The pro-science side has to be rigorously honest. I worry about borderline decisions like the RTS,S malaria vaccine rollout in Africa. The malaria vaccine’s protection is weak, but if parents assume it bulletproofs their kids and become less vigilant, and deaths in vaccinated villages shoot up, it will be a fiasco. The strong Ebola vaccine, on the other hand, appears to be doing pretty well in Africa.

Historically, good science usually beats the fear-mongers. Today’s crisis stems from a diagnostic failure: the surge in autism is real, and parents are legitimately scared. Vaccines aren’t the cause, but some won’t be convinced until we find the cause. We’re roughly where we were in 1982 with AIDS. We didn’t know why gay men were dying, which left room for the crazy hypothesis that it was disco, anal sex, and poppers. That belief died out, but a variant (parasites, bad water, and malaria cause AIDS) clung on into the 1990’s in Africa mostly because of suspicion that Big Pharma was using the epidemic to get rich. Big Pharma is still using the AIDS epidemic to get rich (viz the Truvada struggle).^5^ That breeds conspiracy theories.

**What’s your personal take on non-medical exemptions and the possibility of enforced vaccination of U.S. children?**

The longer I cover medicine, the more of a public-health fascist I become. Nothing in the Bill of Rights enshrines my right to transmit a fatal disease. Nor to say “every other child must be vaccinated so that mine is protected — but no vaccine for my precious darling because he’s special.” So I favor zero non-medical exemptions and compulsory vaccination during outbreaks. The Supreme Court agrees.

So do major religious authorities.

**How have attitudes towards the anti-vax movement recently shifted within the press?**

Journalists – certainly New York Times science journalists – are hardening our attitudes.

Back when the Lancet first broached the possibility of a measles vax link to autism,^6^ we covered it intensively and quoted its proponents respectfully.

But as dissuasive evidence mounted and their scientists were exposed as frauds, they doubled down, claiming all vaccines were dangerous. They shifted theories, first blaming gut leakage, then thimerosal, then immune overload. Eventually, we began viewing them as we do Holocaust denialists and people who insist the moon landing was faked. We don’t feel journalistically obligated to print their “side” any more.

Now I view the movement’s leaders as flat-out predators. Some of them sell worthless supplements or vitamins – or even dangerous ‘treatments’ like chelation. I sympathize with worried parents, but I see them as victims. In 2009, I covered what appeared to be a high rate of autism among Somalis in Minneapolis. The anti-vaxxers leapt in, their high vaccination rates plummeted. By 2017, the Somalis had a major measles outbreak.

**Describe hate mail to reporters like yourself and the related issue of cyber-bullying of pro-vaccine doctors.**

Compared to the threats that doctors like Paul Offit and Peter Hotez get, mine are like valentines.

The most vivid one I’ve received recently was this: “You are a pathetic american. I am going to debunk all of your work, you are a big pharma yes man, your pro vaccines cause the flu and death, speaking of senior citizens, over 30,000 die a year from pharma medicine...your work devalues the whole ny times, and in the future you will be seen as a traitor to US for pushing soft kill weapons with rubbish writtings. Why dont you tell the truth you piece of shit... Zika virus is sold by the rockefeller foundation on the internet! Where is your information on CDC whistleblowers confirmed Wakefields findings that he was black balled for... over 20 years of research into this subject you are hurting America.. you have the brain of a stegasouras and your opinion is social dinosaurism.”

Most writers aren’t as colorful, but the gist of his argument is the usual: Wakefield is a hero, I’m a Big Pharma dupe. If you read all my coverage of Big Pharma, you’d think otherwise.

I try to laugh it off. President Trump’s attacks on us are scarier, but I have a t-shirt saying “Enemy of the People” in the Times’ font.

## Footnotes

1 After a measles outbreak that started in Disneyland in 2014 led to more than 125 cases, California – which previously allowed broad “personal belief” exemptions – outlawed exemptions for anything other than medical reasons. Vaccination rates among kindergarteners rose sharply.

2 In 2007, Jenny McCarthy, a model and actress, wrote a book insisting that vaccines caused her son’s autism; her appearances on “Oprah” and elsewhere brought many mothers into the anti-vaccine fold.

3 In 1955, a bad batch of polio vaccine from Cutter Laboratories, one of several companies making the Salk vaccine, infected thousands of children with live virus. Nearly 200 were paralyzed and 10 died.

4 Starting in 2016, more than 800,000 schoolchildren in the Philippines were given Sanofi’s new dengue vaccine, Dengvaxia. Although a WHO panel had ruled the vaccine safe, it may have made some children more likely to die if they later were infected with a different dengue strain. The government blamed the vaccine for the deaths of 19 children, parents panicked, uptake rates for all vaccines fell and measles cases soared. The vaccine is now limited for use only in people who have had dengue once; in the Philippines, criminal charges are pending.

5 Truvada, a drug that can prevent HIV infection, costs almost $20,000 a year. Activists have been trying to get the government – which paid for the research proving Truvada could do that – to cancel the patent and make the drug available cheaply to the estimated 1 million Americans who need it. Although several generic drug companies have applied to the Food and Drug Administration for the right to sell cheaper versions, the drug’s maker, Gilead Sciences, has sued each one and reached secret out-of-court settlements to delay their market entry.

6 The false rumor that measles vaccine causes autism was triggered by a 1998 article in the Lancet by a British doctor, Andrew Wakefield. Wakefield’s thesis has been disproven by many large studies, his co-authors backed out, the Lancet has retracted the article, and Mr. Wakefield, who was revealed to be working with lawyers suing vaccine companies, lost his medical license. But he remains a hero to anti-vaccine activists.
